# Structure and Aromaticity of Si_3_–Si_7_ Analogues of Fully Conjugated C_3_–C_7_ Aromatic Carbocycles

**DOI:** 10.3390/ijms27073333

**Published:** 2026-04-07

**Authors:** Bagrat A. Shainyan

**Affiliations:** A.E. Favorsky Irkutsk Institute of Chemistry, Siberian Division of the Russian Academy of Sciences, 1 Favorsky Street, 664033 Irkutsk, Russia; bagrat@irioch.irk.ru

**Keywords:** Si_3_–Si_7_ unsaturated cycles, aromaticity, structure, ASE, NICS, aromaticity, MP2 and CCSD calculations, solvent effect

## Abstract

The persilylated Si_3_–Si_7_ analogues of the C_3_–C_7_ aromatic molecules and ions with all hydrogen or all fluorine atoms at silicon have been calculated at high levels of theory, up to MP2/aug-cc-pVTZ for all species and CCSD/6-311++G** for Si_3_ and Si_4_ species, both in the gas phase and in a polar solvent (water). The aromaticity of the calculated species was estimated using structural, energetic, and NMR criteria. (SiF)_3_^+^ cations are more aromatic than (SiH)_3_^+^ by the NICS (nuclear-independent chemoical shift) but less aromatic by the ASE (aromatic stabilization energy) criterion. Dications (SiX)_4_^2+^ are planar (X = H) or slightly puckered (X = F); the ASE decreases by 4–5 kcal/mol upon going from gas to solution, or from X = H to X = F. Dianions (SiX)_4_^2−^are nonplanar and antiaromatic. The ASE for the slightly distorted-from-planarity anion Si_5_H_5_^−^ is ~53 kcal/mol, vs. 85 kcal/mol for its carbon analogue. The structure of Si_6_X_6_ molecules strongly depends on the level of calculations. The NICS and ASE values have been calculated for planar Si_6_H_6_ and (SiH)_7_^+^ but not for strongly distorted Si_6_F_6_ and (SiF)_7_^+^ species.

## 1. Introduction

The structure and properties of silicon analogues of aromatic carbocycles cannot be simply deduced from those of the latter. Even the structure is often principally different, to say nothing of the aromaticity assessed by different criteria. As stated in an earlier work, due to the electropositivity of silicon, the silyl analogues of carbocations should be more stable [1]. For example, (SiH)_3_^+^ was studied theoretically and shown to be the global minimum on the potential energy surface (PES) [1,2]. Moreover, it was also detected experimentally [3]. But, even if we accept this assertion on the relative stability of Si-analogues of carbocations, it can hardly be unreservedly transferred to their aromaticity.

In an early work on the semiempirical [4] and ab initio calculations [5] of dications and dianions of tetrasilacyclobutadiene (SiH)_4_, both were shown to be nonplanar and, hence, nonaromatic. However, much later, the heavily tetrasubstituted tetrasilacyclobutadiene dication was synthesized and fully characterized as the tetraphenylborate salt [6]. The authors also performed DFT calculations clearly confirming its classical 2π-aromaticity. In contrast, the 6π-electron (SiR)_4_^2−^ dianion (R = SiMe*^t^*Bu_2_) [7,8] is not aromatic since it has a strongly puckered ring and nonequivalent Si−Si bonds. The calculations also revealed the absence of a diatropic ring current, confirming its nonaromaticity using the NMR criterion of aromaticity. For the neutral molecule Si_4_H_4_, the global minimum on the PES was recently found to correspond to the puckered Si_4_ ring of *D*_2d_ symmetry, the planar conformer of *D*_4h_ symmetry lying 30.1 kcal/mol higher at the RI-MP2/cc-pVTZ level [9].

Delocalization of the charge is an effective way to stabilize tricoordinated silyl cations [10]. Taking into account that a planar cation (SiH)_3_^+^ and a nearly planar dication (SiH)_4_^+2^ exhibit substantial stabilization energies [1], the question was posed whether the cation (SiH)_5_^+^ can also benefit from similar delocalization [10]. Sometime before this question was asked, only pyramidal *C*_4v_ and *C*_S_ forms were reported as minima on the PES of (SiH)_5_^+^ [11]. Among many other calculated structures [10], the planar (SiH)_5_^+^ cation of *C*_5h_ symmetry has also been calculated but it did not correspond to the energy minimum.

**Figure 1:**
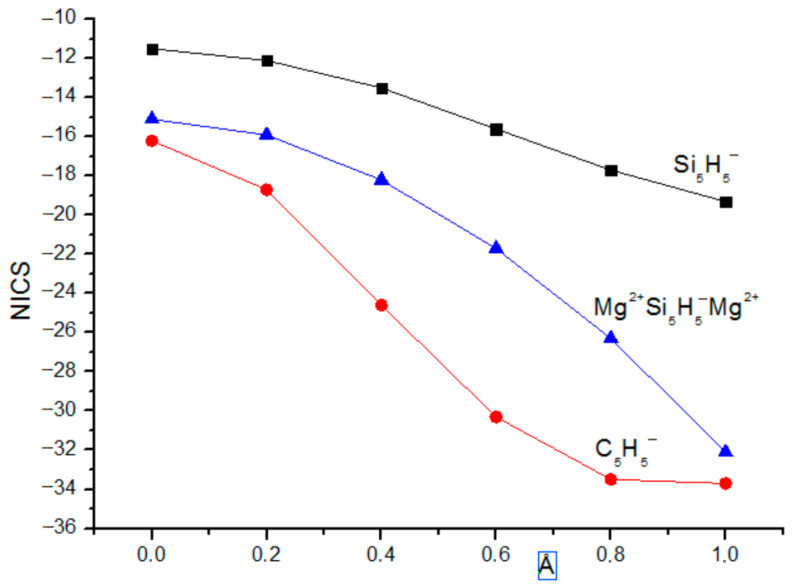
B3LYP/6-311++G** calculated NICS values of Si_5_H_5_^−^, C_5_H_5_^−^ ions and Mg^2+^Si_5_H_5_^−^Mg^2+^ complex.

Unlike the (SiH)_5_^+^ cation, the (SiH)_5_^−^ anion, similar to its carbon predecessor, the well-known cyclopentadienyl anion, is aromatic in planar form, which, however, was a local rather than the global minimum on the PES [12]. The global minimum of (SiH)_5_^−^ is nonplanar, so the pseudo-Jahn–Teller effect overcomes the stabilization due to the aromaticity of the planar form. For the planar (SiH)_5_^−^, as well as for its carbon analogue and the Mg^2+^Si_5_H_5_^−^Mg^2+^ complex as reference structures, the calculated values of the NICS are negative [12], as shown in Figure 1, being indicative of the aromaticity of the (SiH)_5_^−^ anion. An independent indication of aromaticity is equalization of the Si–Si bonds in (SiH)_5_^−^. Less negative NICS values for (SiH)_5_^−^ than for (CH)_5_^−^ suggest a lower aromaticity of the former. In the magnesium complex, the planarity of the (SiH)_5_^−^ fragment is restored, making the complex more aromatic than the anion itself though less aromatic than the (CH)_5_^−^ anion (Figure 1).

A similar conclusion about the lower aromaticity of the (SiH)_5_^−^ motif can be deduced from the charge distribution in the persilasubstituted ferrocene, (Si_5_H_5_)_2_Fe, investigated theoretically in an early work [13] (published before the NICS values were proposed as a criterion of aromaticity [14]). The positive charge on the metal (and, hence, half of it with the opposite sign on each Si_5_H_5_ fragment) in the most extended basis is almost twice as low as that in ferrocene itself: −0.26 on Si_5_H_5_ versus −0.44 on C_5_H_5_. Note that the negative charge on Si_5_H_5_ is fixed mainly on hydrogen atoms, whereas on C_5_H_5_ it is located on carbon atoms. Very recently and practically at the same time, two works appeared in which penrasilacyclopentadienide alkali metal salts were obtained and characterized [15,16]. The aromaticity of the salt Si_5_R_5_^−^Li^+^ (R = 2,4,6-triisopropylphenyl) [15] does not characterize the Si_5_ ring but is rather akin to the spatial aromaticity in the dilithium salts of tetrasilylcyclobutadiene dianions [17], the more so that the authors themselves emphasize the pivotal role of the lithium cation stabilizing the salt [15]. The aromaticity was also confirmed by the M06-2X/def2-SVP calculated values of the NICS in the center of the Si_5_ ring, reaching −19 ppm. An unexpected statement in [15] that deserves special consideration is that the very high flexibility of the studied salt questions the seemingly immutable tenet of a clear-cut distinction between resonance and equilibrium. Lithium and potassium salts with the same counter-ion Si_5_R_5_^−^ were prepared in a higher yield and analyzed by X-ray as crystalline solvates with THF or 2,6-dimethylphenyl isocyanide [16]. With the latter ligand, the authors succeeded in preparing single crystals without disordering the lithium atom located 2.082 Å from the center of the mean plane of the Si_5_ ring. The calculation at the M06-2X/def2-TZVP-level planar Si_5_ ring is a local minimum lying 7.43 kcal/mol above the puckered structure, which is the global minimum on the PES [16]. The NICS values calculated at the same level of theory are equal to −11.5 ppm for Si_5_H_5_^−^ and −16.3 ppm for its congener C_5_H_5_^−^, indicating the lower aromaticity of the former.

In an early study [18], Si_6_H_6_ was shown to be a local minimum on the PES corresponding to a planar structure of *D*_6h_ symmetry at the HF level of theory with 3-21G(d,p), HF/6-31G, and 6-311G(d,p) basis sets. However, the optimized structure is strongly dependent on the level of theory. Thus, at the HF/6-311++G(d,p) level, the Si_6_H_6_ molecule is nonplanar and distorted to chair, with an angle between the Si1Si2Si6 and Si2Si3Si5Si6 planes of 15° and SiSiSi angles of 119.3°, indicating almost trigonal-planar arrangement at the silicon atoms. At the MP2/cc-pVTZ level, the molecule becomes even more strongly distorted, with an angle between the Si1Si2Si6 and Si2Si3Si5Si6 planes of 27° and the SiSiSi angles reduced to 117.4°. The calculated NICS(0) value is −11.32 ppm. However, at the MP2/aug-cc-pVTZ level, the Si_6_H_6_ molecule is planar again and has *D*_6h_ symmetry, with all SiSiSi angles of 120°, all Si–Si bonds equal to 2.214 Å, and the value of the NICS(0) equal to −12.91 ppm, attesting to its higher aromaticity.

Remarkably, both planar and chair hexasilabenzene Si_6_H_6_ correspond to local minima on the PES, whereas the global minimum corresponds to hexasilaprismane, an analogue of the Ladenburg benzene (prismane) proposed as early as in 1869 as an alternative to the Kekulé benzene, also allowing for an explanation of the equivalence of all CH groups (but not the number of *ortho*-, *meta*-, and *para*-isomers) [19]. Prismane was synthesized more than a century later in only a 1.8% yield from benzvalene by the photolysis of its azo derivative [20]. It is stable at room temperature but upon heating is transformed to benzene. Hexasilaprismane was obtained by Sekiguchi after another 20 years in an even lower yield (1%) by dechlorination of 1,2-bis-(2,6-diisopropylphenyl)-1,1,2,2-tetrachlorodisilane with magnesium in THF [21]. While the molecule of prismane was estimated to be 90 kcal/mol less stable than benzene (although the barrier to isomerization was also very high [22]), hexasilaprismane was calculated to be −6.7 and −9.5 kcal more stable than hexasilabenzene at the HF/3-21G and HF/6-31G*//HF/3-21G levels, respectively [18]. Our high-level calculations in this work at the CCSD(T)/cc-pVTZ//MP2/aug-cc-pVTZ level of theory (vide infra) gave an even higher stability of hexasilaprismane with respect to hexasilabenzene, equal to −16.4 kcal/mol.

With all this in mind and taking into account the dependence of the results of calculations on the level of theory, in the present work, we calculated the persilylated Si_3_–Si_7_ analogues of the C_3_–C_7_ aromatic molecules with hydrogen and fluorine atoms at silicon in the neutral and ionic forms at a high level of theory. It should be noted that the effect of silicon atoms replacing carbon atoms in the ring of aromatic hydrocarbons is principally different from that of silyl groups as substituents at the carbon atoms in the same rings, since in aromatic hydrocarbons the diatropic current flows around the CH moieties, whereas in S-analogues it circulates around the SiH moieties.

## 2. Results and Discussion

### 2.1. Si_3_ Cycles

The results of the MP2/cc-pVTZ and, for comparison, CCSD/6-311++G** calculations and assessment of aromaticity of the Si_3_-cycles are summarized in Table 1. The aromatic stabilization energies (ASEs) were calculated as the energies of the hyperhomodesmotic reaction (1) by analogy with that for the carbon analogues [23]:




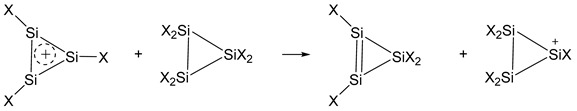

(1)


All calculated structures are fully planar; that is, all X atoms at the three-coordinated silicon atoms lie in the plane of the ring. The Si–Si bonds in the case of X = F are longer than those for X = H because of repulsion of more positively charged silicon atoms in the former. Note also that they are slightly shorter in solution, in agreement with what one would expect for more polar structures in a polar medium.

**Table 1:** Structures, Si–Si bond lengths (Å), total charges on ring (*e*), and ASEs (kcal/mol) for trisilacyclopropene cation. MP2/cc-pVTZ results are given as plain text, and CCSD/6-311++G** results are given in italics.

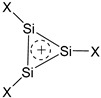										
X	Gas					PCM (ε = 83)				
	Structure	*l*_Si–Si_	Σ*q*_Si_	|*q*_Si_ − *q*_X_|	ASE	Structure	*l*_Si–Si_	Σ*q*_Si_	|*q*_Si_ − *q*_X_|	ASE
H	Full planar	2.207	0.632	0.078	−23.94	Full planar	2.202	0.729	0.153	−23.85
*H*	*Full planar*	*2.193*	*0.942*	*0.295*	*−24.43*	*Full planar*	*2.188*	*1.053*	*0.368*	*−26.61*
F	Full planar	2.243	1.750	0.833	−11.71	Full planar	2.235	1.890	0.927	−11.26
*F*	*Full planar*	*2.215*	*1.476*	*0.651*	*−12.40*	*Full planar*	*2.207*	*1.647*	*0.765*	*−11.15*

Both methods indicate lowering of aromaticity in going from X = H to X = F, with a very small difference between the methods. At the CCSD level, in both media, all calculated species are more compressed, as evident from the Si–Si bond lengths in the Si_3_X_3_^+^ cations, which are shorter by 0.014–0.028 Å. The same is true for all other species in the hyperhomodesmotic reaction (1). A more tight CCSD structure also results in a larger polarity of the Si–H bonds, |*q*_Si_ − *q*_H_|, both in gas and in solution (Table 1). On the contrary, the polarity of the Si–F bonds, |*q*_Si_ − *q*_F_|, at the CCSD level in both media is somewhat lower. Apparently, this is due to the same sign of the charge on the Si and H atoms and sharply differing opposite signs on the Si and F atoms. Therefore, the choice of the method is not critical for the analysis of the aromaticity of the studied systems.

**Figure 2:**
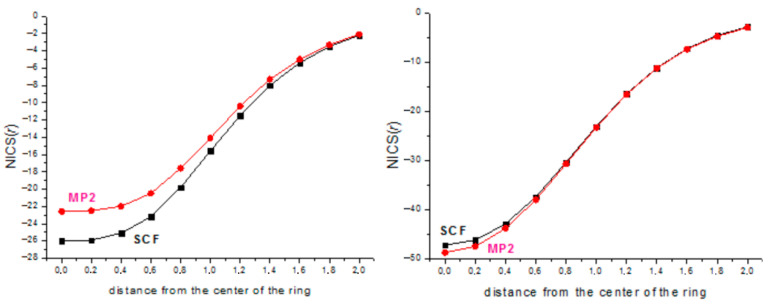
NICS(*r*) of (SiH)_3_^+^ cation (**left**) and (SiF)_3_^+^ cation (**right**).

The values of the nuclear-independent chemical shifts from NICS(0) to NICS(2.0) with the step of 0.2 Å are plotted in Figure 2.

The practically equal values of ASEs in gas and solution in Table 1 clearly demonstrate that the aromatic stabilization energy obtained by using the properly chosen hyperhomodesmotic reaction is a characteristic inherent to the process and independent of external effects. The substituent effect (X = H, F) is the most pronounced in or near the center of the ring and decreases with the distance from the center. For X = H, the SCF-calculated NICS values are less negative than those at the MP2 level, merging only at *r* ~ 2.0 Å, whereas for X = F, the order of the SCF and MP2 NICS values is the opposite at small *r* distances and practically disappears at *r* ≥ 0.6 Å. Overall, by the NMR criterion, the (SiF)_3_^+^ cation is more aromatic, especially closer to the ring plane, apparently, due to the *n*_F_ → π* conjugation increasing the π-electron density on the ring. In contrast, the energetic criterion (ASE) (Table 1) indicates that the aromaticity of the (SiF)_3_ cation is twice as low as that of the (SiH)_3_ cation. This is fully consistent with the conclusion that the ASE values strongly depend on the substituent being larger for electron-donating groups increasing the aromaticity [24,25].

**Figure 3:**
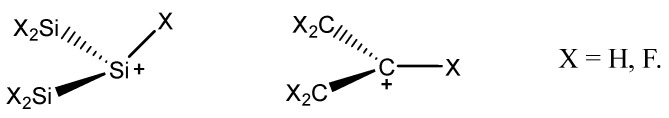
Schematic structures of nonplanar [Si_3_X_5_]^+^ and planar [C_3_X_5_]^+^ cations.

A noteworthy difference is observed between the [Si_3_X_5_]^+^ cations in reaction (1) and their carbon analogues [C_3_X_5_]^+^. Both for X = H and F, in gas and in solution, the optimized structures correspond to the open angular cations [X_2_Si–Si(X)–SiX_2_]^+^ rather than to the cyclic structures, with the Si–X bonds forming an angle of ~100° with the Si_3_ plane (Figure 3). Although in [C_3_X_5_]^+^ cations the X_2_C–CX_2_ bond is also ruptured during optimization, they remain planar (Figure 3).

Therefore, the (SiX)_3_^+^ cations (X = H, F) are fully (including substituents X) planar aromatic species, being more aromatic for X = F from the NMR criterion (NICS) but less aromatic from the energy criterion (ASE), both at the MP2 and CCSD levels of theory.

### 2.2. Si_4_ Cycles

As mentioned in the Introduction, early calculations showed (SiH)_4_^2+^ and (SiH)_4_^2−^ to be nonaromatic [4,5]. The tetrasubstituted tetrasilacyclobutadiene dication (SiR)_4_^2+^ (R = PhC(N(*^t^*Bu)–)_2_, obtained as the tetraphenylborate salt, is only slightly puckered and is aromatic [6]. Our recent MP2/cc-pVTZ calculations of (Si_4_H_3_)CH=C(CN)_2_ with the potentially aromatic (Si_4_H_3_)^2+^ dicationic residue in the molecule showed that in the gas phase, it is rearranged with cyclization. In a polar solvent, the planar structure is retained but the Si–Si bonds are far from being equal and the NICS values are positive, indicating antiaromaticity of the Si_4_ cycle in this molecule [26].

**Figure 4:**
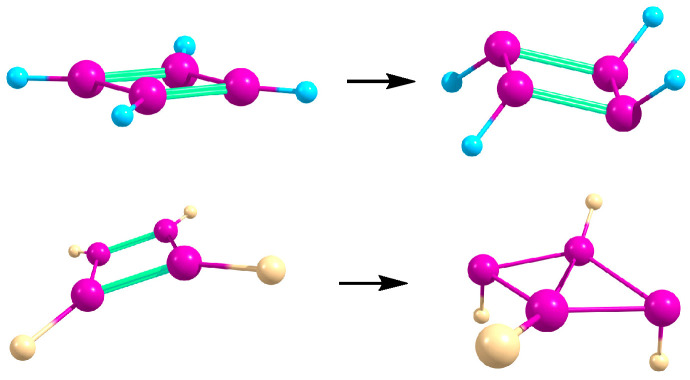
Distortion of (SiH)_4_^2−^ and (SiF)_4_^2−^ dianions during optimization.

As distinct from the earlier semiempirical MINDO/3 calculations [4], our high-level MP2/cc-pVTZ calculations allow for localization of the square-planar structure of dications [SiX_4_]^2+^ as local minima on the PES. However, as in the early work [4], the corresponding dianions (SiX)_4_^2−^ were distorted during optimization (Figure 4), so they are antiaromatic and were excluded from further consideration.

The structure, Mulliken atomic charges and ASE values for dications (SiH)_4_^2+^ and (SiF)_4_^2+^ calculated at the MP2/cc-pVTZ and, for comparison, CCSD/6-311++G** levels are summarized in Table 2. The ASE values were calculated as the energies of the hyperhomodesmotic reaction (2) by analogy with that for the carbon analogues [23]:




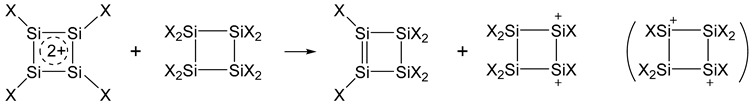

(2)


**Figure 5:**
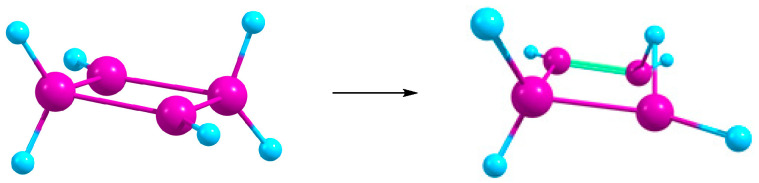
The optimized ‘open-envelope’ conformation of the Si_4_H_6_^2+^ dication starting from the planar 1,3-dication.

Note that while the ring in the molecule of tetrasilacyclobutane Si_4_H_8_ calculated at both levels of theory has a folded structure with puckering angles of 29.0° or 24.5° in gas and 27.9° or 22.7° in solution, the ring in its fluorinated analogue Si_4_F_8_ is planar in both media. The ring in the molecule of tetrasilacyclobutene Si_4_H_6_ is planar, with HSi=SiH, HSi–SiH_2_ and H_2_Si–SiH_2_ bonds of 2.165, 2.319, and 2.365 Å in both media. Very close geometry was obtained at the CCSD level. In contrast, the ring in the Si_4_F_6_ molecule is folded, with puckering angles of 12.9° in gas and 13.9° in solution. The FSi = SiF, FSi–SiF_2_, and F_2_Si–SiF_2_ bond distances are 2.173, 2.298, and 2.346 Å in gas and 2.174, 2.291, and 2.341 Å in solution. More complicated for the hyperhomodesmotic reaction (2) is the choice between 1,2- and 1,3-dications Si_4_X_6_^2+^. On the one hand, in 1,3-dications, the charges are spatially separated, so they should be more stable, and, indeed, they lie ~3 kcal/mol lower in energy. On the other hand, the structure of 1,2-dications is closer to the model tetrasilacyclobutenes Si_4_X_6_ that is the principal for hyperhomodesmotic reactions. Moreover, for X = H, the structure of 1,3-dications is not retained during optimization in gas: one of the SiH_2_ hydrogens moves to the adjacent tricoordinated silicon atom, forming the drastically distorted structure (Figure 5).

Therefore, for estimation of the ASEs, 1,2-dications [Si_4_X_6_]^2+^ were used. Dication [Si_4_H_6_]^2+^ is folded both in gas and in solution, with puckering angles of 20.0 and 12.8°, respectively. The SiSi bonds are almost equal, ~2.40 Å. Dication [Si_4_F_6_]^2+^ is planar in gas and slightly folded in solution (3.5°). The FSi = SiF, FSi–SiF_2_, and F_2_Si–SiF_2_ bonds are 2.488, 2.512, and 2.411 Å in gas and 2.441, 2.458, and 2.405 Å in solution.

**Table 2:** Structures, Si–Si bond lengths (Å), total charges on ring (*e*), and ASEs (kcal/mol) for (SiX)_4_^2+^ dications. MP2/cc-pVTZ results are given as plain text, and CCSD/6-311++G** results are given in italics.

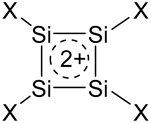								
X	Gas				PCM (ε = 83)			
	Puckering Angle	*l*_Si–Si_	Σ*q*_Si_	ASE	Puckering Angle	*l*_Si–Si_	Σ*q*_Si_	ASE
H	0° (planar)	2.273	1.328	−44.11	0° (planar)	2.252	1.576	−39.89
*H*	*12.4°*	*2.269*	*1.284*	*−39.32*	*12.7°*	*2.248*	*1.948*	*−33.52*
F	19.1°	2.280	2.768	−40.09	18.5°	2.253	3.044	−35.20
*F*	*26.1°*	*2.315*	*2.420*	*−38.93*	*29.3°*	*2.284*	*2.700*	*–*

In the gas phase, dications (SiX)_4_^2+^ calculated at the MP2/cc-pVTZ level of theory are square-planar ((SiH)_4_^2+^) or somewhat puckered ((SiF)_4_^2+^), with Si–Si bond lengths of 2.273 and 2.280 Å, respectively. In solution, the Si–Si bonds become shorter and practically equal at 2.252 and 2.253 Å. At the CCSD/6-311++G** level, all (SiX)_4_^2+^ dications are puckered both in gas and in solution, with the degree of puckering being notably larger for X = F than for X = H (Table 2).

In the gas phase, in both methods, about two-thirds of the formal positive charge in (SiH)_4_^2+^ is located on the silicon atoms, whereas in solution, the localization is increased, especially at the CCSD/6-311++G** level. For (SiF)_4_^2+^, the total charge on the silicon atoms exceeds the formal positive charge of +2, varying from +2.4 to +3.0 on the silicon atoms.

As follows from Table 2, the aromatic stabilization energy (ASE) decreases by 4–5 kcal/mol upon going from gas to solution. This is consistent with weakening the role of internal factors (intramolecular polar interactions) in a polar medium and the lower aromaticity of fluorinated species [27]. The ASE values are also somewhat lower at the CCSD level than at the MP2 level for X = H and very close to it for X = F in gas. In solution, the ASE for X = F was not calculated because (i) the CCSD optimization failed to reach convergence and (ii) the (SiF)_4_^2+^ dication is strongly distorted, which largely deprives the calculation of practical value.

**Figure 6:**
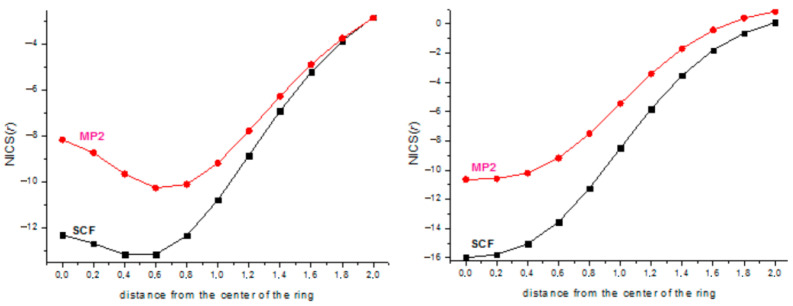
NICS(*r*) of (SiH)_4_^2+^ dication (**left**) and (SiF)_4_^2+^ dication (**right**).

In Figure 6, the NICS values for the (SiH)_4_^2+^ and (SiF)_4_^2+^ dications calculated at the MP2/cc-pVTZ level of theory are plotted. The different profiles of the curves are probably due to the fully planar geometry of the former and the slightly puckered geometry of the latter.

Consequently, as in the case of the Si_3_ cycles above, the choice of method is not critical for the analysis of the aromaticity of Si_4_ systems.

### 2.3. Si_5_ Cycles

**Figure 7:**
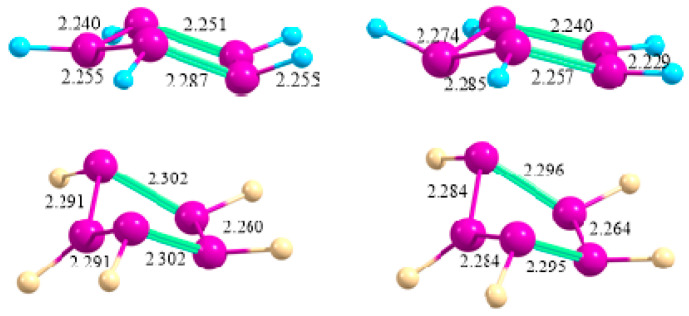
The structures of the Si_5_H_5_^−^ (**top**) and Si_5_F_5_^−^ (**bottom**) anions in gas (**left**) and in a polar medium (**right**) with the Si–Si bond lengths (Å).

At the MP2/6-31G* level of theory, only two energy minima of *C*_S_ and *C*_2_ symmetries were located on the potential energy surface of the persilacyclopentadienyl anion, having very close energies and lying 8.3 kcal/mol lower than the planar aromatic Si_5_H_5_^−^ anion of *D*_5h_ symmetry [1]. The latter form was considered as aromatic according to the molecular orbital chemical bonding analysis and Si–Si bond equalization [28]. However, at the B3LYP/6-311++G** and CCSD(T)/6-311++G** levels of theory [28], as well as in our MP2/cc-pVTZ and CCSD/6-311++G** calculations in the present work, it was shown to be nonplanar. The latter structures of the Si_5_H_5_^−^ and Si_5_F_5_^−^ anions in gas and in polar medium are presented in Figure 7.

It does not make sense to calculate the values of the ASE or NICS for such strongly distorted nonaromatic structures, although in an early work, the value of the ASE for the Si_5_H_5_^−^ anion was estimated to be 52.75 kcal/mol, vs. 84.67 kcal/mol for its carbon analogue [1].

### 2.4. Si_6_ Cycles

The structure of Si_6_H_6_ strongly depends on the level of calculations. In an early work, it was shown to be planar at the HF/3-21G**, HF/6-31G, and HF/6-311G** levels [18]. At the HF/6-311++G** and MP2/cc-pVTZ levels, the ring is distorted to the chair. However, at the MP2/aug-cc-pVTZ level, the Si_6_H_6_ molecule is planar again [29].

**Figure 8:**
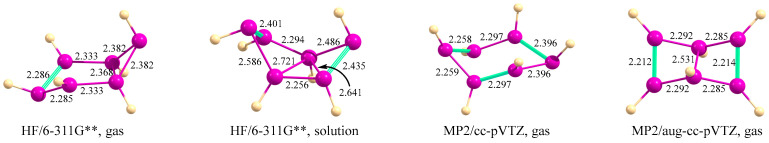
Structure of Si_6_F_6_ optimized at different levels of theory in gas and solution starting with planar hexagonal structure. No convergence could be reached at MP2 level in solution.

Much less clear is the situation with hexafluorohexasilabenzene Si_6_F_6_. In polymeric form, (Si_6_F_6_)_n_, it was supposed to have electronics applications and be stable in the nanosheet form [30]. Theoretical calculations [31,32,33] showed that, unlike planar fluorographene with all sp^2^ carbon atoms, fluorosilicene has a buckled structure with mixed sp^3^-sp^2^ hybridization. As in the case of Si_6_H_6_, the optimized structure of the monomeric Si_6_F_6_ molecule drastically depends both on the method of calculations and the basis set used. In Figure 8, the structures of Si_6_F_6_ at different levels of theory are shown, including 1,2,3,4,5,6-hexafluorobicyclo[3.1.0]persilahexane, 1,2,3,4,5,6-hexafluoro-1λ^5^,2λ^3^-tricyclo-[2.2.0.0^1,3^]hexasil-5-ene, distorted hexafluorohexasilabenzene, and hexafluorobicyclo-[2.2.0]hexasila-2,5-diene. Apparently, the diversity of structures of Si_6_F_6_ is due to repulsion between the fluorine atoms bearing substantial negative charge.

**Figure 9:**
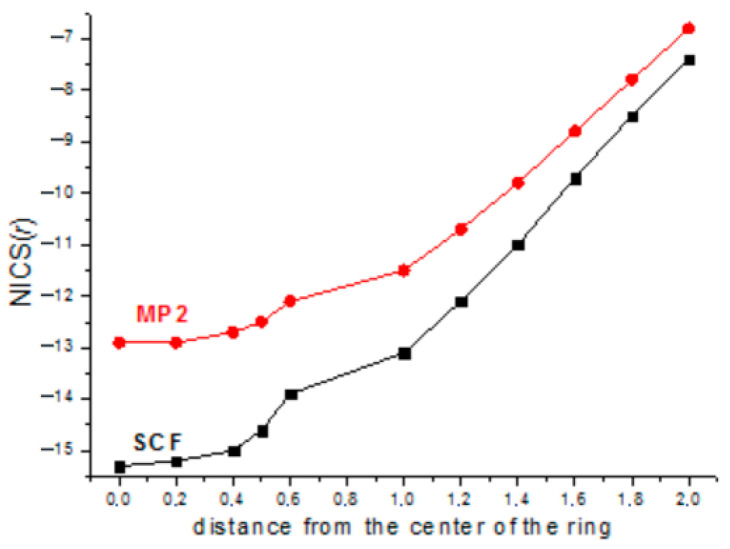
The NICS values calculated at the MP2/cc-pVTZ level of theory on the geometry of Si_6_H_6_ optimized at the MP2/aug-cc-pVTZ level (planar).

With this in mind, we have calculated the NICS values only for hexasilabenzene at the MP2/cc-pVTZ level of theory, but for the planar geometry optimized at the MP2/aug-cc-pVTZ level; the results are depicted in Figure 9.

The aromatic stabilization energy of planar hexasilabenzene was calculated as the energy of the hyperhomodesmotic reaction (3) at the MP2/cc-pVTZ//MP2/aug-cc-pVTZ level in the gas phase:




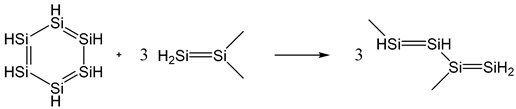

(3)


The Si–Si bond lengths in Si_6_H_6_ are equal to 2.214 Å, the total charge on the silicon atoms (Σ*q*_Si_) is −0.072*e*, and the ASE value is −15.47 kcal/mol.

### 2.5. Si_7_ Cycles

The aromatic stabilization energy of the planar persilatropylium cation (SiH)_7_^+^ was calculated as the energy of the hyperhomodesmotic reaction (4) at the MP2/cc-pVTZ level in gas and in solution; the results are summarized in Table 3.




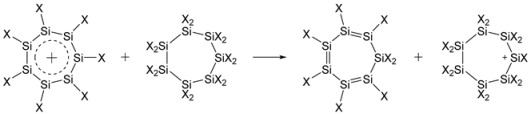

(4)


**Table 3:** Structures, Si–Si bond lengths (Å), total charges on ring (*e*), and ASEs (kcal/mol) for persilatropylium cations (SiX)_7_^+^.

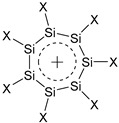						
X	Gas			PCM (ε = 83)		
	*l*_Si–Si_	Σ*q*_Si_	ASE	*l*_Si–Si_	Σ*q*_Si_	ASE
H	2.223	0.475	−25.86	2.219	0.728	−6.44
F	2.229	3.482	–	2.251–2.287	2.916	–

**Figure 10:**
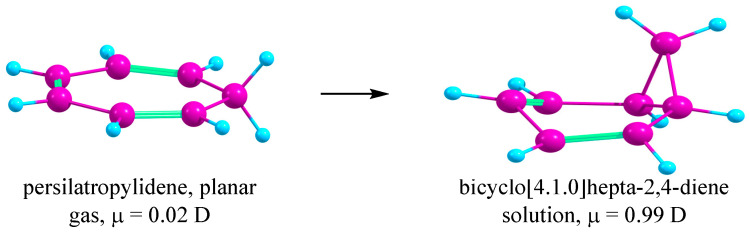
Geometry optimization of planar nonpolar persilatropylidene molecule in solution.

The lower ASE for the persilatropylium cation (SiH)_7_^+^ in solution is due to the conversion of the persilatropylidene molecule, which is planar and nonpolar in gas, to the folded and polar bicyclo [4.1.0]hepta-2,4-diene in solution upon the geometry optimization (Figure 10).

The analysis of the optimization convergence revealed that the folding and bicyclization lower the energy by ~16 kcal/mol, so the external solvent effect itself is rather small (vide supra).

**Figure 11:**
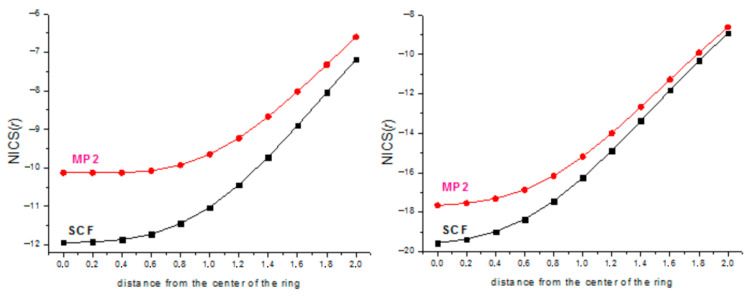
NICS(*r*) of (SiH)_7_^+^ (left) and (SiF)_7_^+^ (planar) cations.

**Figure 12:**
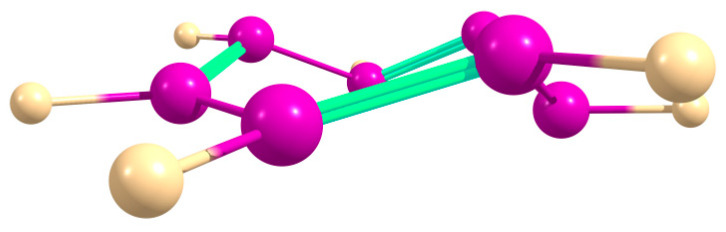
Optimized chair-like structure of (SiF)_7_^+^ cation in gas and in polar medium.

The (SiH)_7_^+^ cation is fully planar, which allowed for calculations of its NICS values (Figure 11). For the (SiF)_7_^+^ cation, the global minimum corresponds to the nonplanar chair-like structure (Figure 12), which made it meaningless to calculate the NICS for this form. However, a shallow planar minimum was also located on the PES, lying ca. 9.5 kcal/mol above the global one; thus, for the purpose of comparison, we have calculated the NICS values for it (Figure 11). As in Figure 2, the NICS values in Figure 11 are indicative of the more aromatic nature of the (SiF)_7_^+^ cation due to the *n*_F_ → π* conjugation increasing the π-electron density on the ring.

The value of the ASE calculated for the planar (SiH)_7_^+^ cation corresponding to a shallow local minimum on the PES is positive at 38.74 kcal/mol. For comparison, for the model planar tropylium cation (CH)_7_^+^, the value of the ASE is −76.88 kcal/mol [34]. Apparently, such a difference is due to the aforementioned fact that in the tropylium ion the diatropic current circulates around the CH moieties, whereas in (SiH)_7_^+^ it circulates around the SiH moieties.

The energetic criterion of aromaticity can hardly be applied to the nonplanar chair-like (SiF)_7_^+^ cation (Figure 12), although the formally calculated ASE is negative at −29.19 kcal/mol. The difference from the positive ASE value for (SiH)_7_^+^ may be due to the *n*_F_ → π* conjugation in the former, increasing the electron density on the silicon rim of the ring (although nonplanar) favoring the ring current.

## 3. Materials and Methods

### Computational Details

Most of the calculations were performed at the MP2/cc-pVTZ level of theory (if not stated otherwise) using the Gaussian09 program package, Revision E. 01 [35] with the full optimization of the geometry without symmetry restrictions. For the Si_3_ and Si_4_ molecules and ions, a near “gold standard” method, CCSD/6-311++G**, was used to verify the results of the MP2 calculations, since in its canonical form the latter is known to overestimate dispersion interactions. The NICS values (nuclear-independent chemical shifts) at the center of the ring and at difference distances in the perpendicular direction were calculated within the GIAO (gauge-including atomic orbital) approximation [36] at the SCF and MP2 levels of theory. The solvent effect was investigated by using the polarizable continuum model PCM [37] and water as a highly polar solvent (ε = 83).

## 4. Conclusions

High-level calculations of the persilylated Si_3_–Si_7_ analogues of the C_3_–C_7_ aromatic molecules and ions with all hydrogens or all fluorine atoms at silicon in the gas phase and in a polar solvent have been performed. The aromaticity of the calculated species was estimated using structural (planarity, equalization of the Si–Si bonds), energetic (aromatic stabilization energy), and NMR (nuclear-independent chemical shift) criteria. The conclusions about aromaticity based on different criteria may contradict each other. Thus, according to more negative NICS values, (SiF)_3_^+^ cations are more aromatic than (SiH)_3_^+^, whereas the ASE values for (SiF)_3_^+^ in gas and solution are twice as low as those for (SiH)_3_^+^. This is in agreement with the statement that the ASE values are larger for electron-donating groups increasing the aromaticity.

Dications (SiX)_4_^2+^ are planar (X = H) or slightly puckered (X = F). Dianions (SiX)_4_^2−^ are nonplanar and antiaromatic. For the Si_4_X_6_^2+^ species, the structure of 1,2- rather than 1,3-dications was chosen for calculation of the energies of the hyperhomodesmotic reactions because the former are structurally closer to the model molecules Si_4_X_6_. The values of the ASEs were found to diminish by 4–5 kcal/mol upon going from gas to solution, or from X = H to X = F.

Because of the nonplanarity of the Si_5_H_5_^−^ and Si_5_F_5_^−^ anions in gas and in solution, their ASE values were not calculated, although in the early work, the value of the ASE for the Si_5_H_5_^−^ anion was estimated to be 52.75, vs. 84.67 kcal/mol for its carbon analogue, the cyclopentadienyl anion.

The structure of Si_6_X_6_ molecules strongly depends on the level of calculations. In this work, the Si_6_H_6_ molecule was found to be planar at the MP2/aug-cc-pVTZ level but distorted to chair at the MP2/cc-pVTZ level, so the NICS and ASE values were calculated at the MP2/cc-pVTZ//MP2/aug-cc-pVTZ level of theory. The Si_6_F_6_ molecule is strongly distorted at all levels of theory.

The persilatropylium cation (SiH)_7_^+^ is planar and aromatic by the NMR and ASE criteria. In solution, the value of the ASE is much lower than that in gas due to the different structure and polarity of the persilatropylidene molecule Si_7_H_8_ as a species in the hyperhomodesmotic reaction. In contrast, for (SiF)_7_^+^, the global minimum corresponds to a chair-like structure. Nevertheless, a shallow planar minimum lying ca. 9.5 kcal/mol above the global one was located, allowing for the calculation of the NICS values for it and showing the (SiF)_7_^+^ cation to be more aromatic than the (SiH)_7_^+^ cation.

## Data Availability

The original contributions presented in this study are included in the article. Further inquiries can be directed to the author.
